# Fracture of the Fore-arm

**Published:** 1842-10-29

**Authors:** 


*Fracture of the Forearm; Gangrene produced by the Application of the Starched Bandage.*—A child, æt. 12, of delicate constitution, fractured both bones of the fore-arm by a fall from a carriage; the surgeon applied the ordinary apparatus, which he had previously moistened with clean water; this tightened application caused horrible sufferings during four days, when the surgeon replaced it by the starched bandage, which was less tight than the first, so that it was more easily borne; nevertheless, two days afterwards, dark vesications showed themselves at the extremities of the fingers, and on the 12th the patient was brought to the Hôtel-Dieu, with well-marked gangrene of the hand and lower part of the fore-arm. On the following day amputation of the fore-arm was performed. The patient ultimately recovered.—*Ibid, from Gazette Médicale de Paris.*

